# Antiproliferative and Structure Activity Relationships of Amaryllidaceae Alkaloids

**DOI:** 10.3390/molecules200813854

**Published:** 2015-07-30

**Authors:** Juan C. Cedrón, Ángel G. Ravelo, Leticia G. León, José M. Padrón, Ana Estévez-Braun

**Affiliations:** 1 Instituto Universitario de Bio-Orgánica Antonio González (CIBICAN), Universidad de La Laguna, Av. Astrofísico Francisco Sánchez 2, La Laguna 38206, Spain; E-Mails: jccedron@hotmail.com (J.C.C.); agravelo@ull.es (A.G.R.); lgleon@ull.edu.es (L.G.L.); jmpadron@ull.es (J.M.P.); 2 Departamento de Ingeniería Química Industrial, Universidad de Ingeniería & Tecnología (UTEC), Av. Cascanueces 2221 Santa Anita, Lima 43, Peru; 3 Departamento de Química Orgánica, Universidad de La Laguna, La Laguna 38206, Spain

**Keywords:** Amarylidaceae alkaloids, antiproliferative activity, SAR

## Abstract

The antiproliferative activity of a set of seven natural Amaryllidaceae alkaloids and 32 derivatives against four cancer cell lines (A2780, SW1573, T47-D and WiDr) was determined. The best antiproliferative activities were achieved with alkaloids derived from pancracine (**2**), haemanthamine (**6**) and haemantidine (**7**). For each skeleton, some structure-activity relationships were outlined.

## 1. Introduction

Cancer is a major health problem all over the world. It is responsible of the death of over 8 million people every year, and almost 600,000 deaths in the United States, during 2014 [1]. Alkaloids, such as paclitaxel, vincristine or vinblastine, are known by possessing important antitumor properties and have been used in the last years for the treatment of cancer [2].

The Amaryllidaceae alkaloids have gained much interest because of their wide range of biological activities. For example, acetylcholinesterase [3], analgesic [4], antifungic [5] and antimalarial [6,7,8] activities have been reported for these alkaloids. Since the isolation of pancratistatin [9], a narciclasine-type alkaloid, and the discovery of its important antitumor properties [10], representative alkaloids of this family have been evaluated as potential cytotoxic agents [11]. More recently, some studies focus on the potential as anticancer agents of semisynthetic derivatives of lycorine [12], narciclasine [13] and crinine [14] have also been performed.

As a part of our ongoing research on *Pancratium* alkaloids, this work reports the antiproliferative activity of some Amaryllidaceae alkaloids, and semisynthetic derivatives with pancracine, homolycorine and haemanthamine skeletons, against four human tumor cell lines (A2780 ovary, SW1573 lung, T-47D breast and WiDr colon). Some structure-activity relationships are also presented.

## 2. Results and Discussion

**Figure 1:**
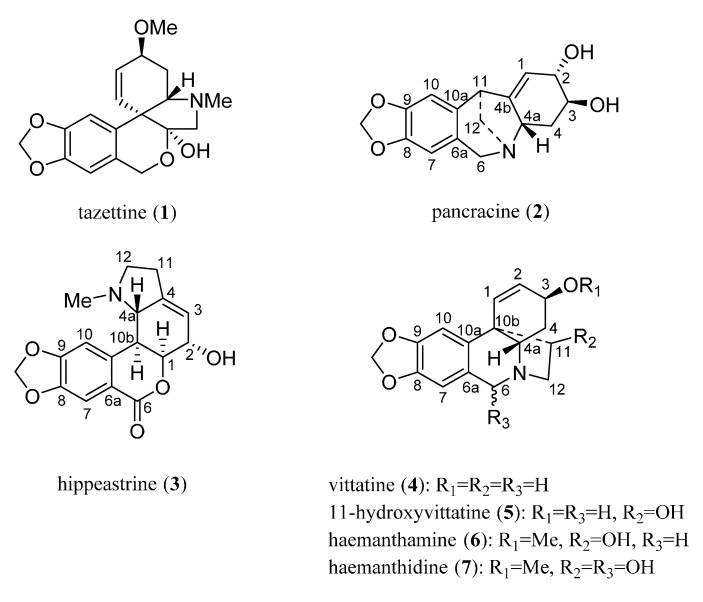
Structure of Amaryllidaceae alkaloids from *Pancratium canariense*.

Fresh bulbs from *Pancratium canariense* were chopped and macerated with MeOH for two weeks at room temperature. The bulbs were filtered, dried, and powdered for a second extraction, using a Soxhlet apparatus with MeOH. Both extracts were collected, concentrated and treated as described [15]. Tazettine (**1**) (3 mg), pancracine (**2**) (17 mg), hippeastrine (**3**) (1.35 g), vittatine (**4**) (8 mg), 11-hydroxyvittatine **5** (123 mg), haemanthamine **6** (2.01 g) and haemanthidine **7** (360 mg) were isolated (Figure 1).

Several derivatives from the major alkaloids hippeastrine (**3**), 11-hydroxyvittatine (**5**), haemanthamine (**6**) and haemanthidine (**7**) were prepared with the aim to generate structurally diverse compounds [6,7,8].

**Scheme 1:**
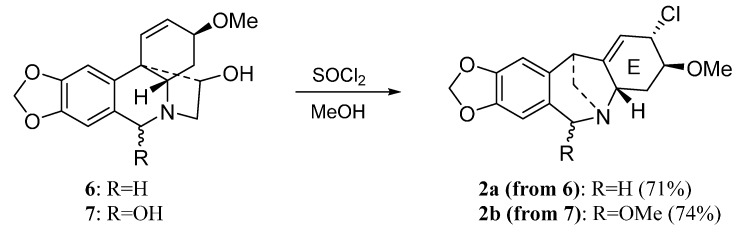
Preparation of montanine-type derivatives **2a** and **2b**.

Scheme 1 shows the preparation of montanine-type derivatives **2a** and **2b** by thionyl chloride mediated rearrangement of **6** and **7**, respectively [16].

**Scheme 2:**
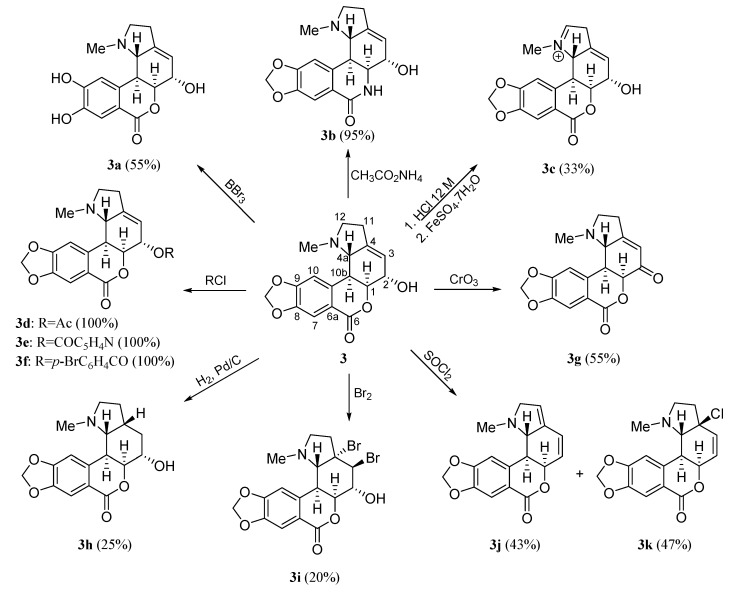
Preparation of homolycorine-type derivatives **3a**–**3k**.

Scheme 2 summarizes the modifications achieved on the structure of the homolycorine-type alkaloid hippeastrine **3** [7]. Derivatives were obtained by modifying the methylendioxy group (**3a**), the lactone moiety (**3b**), the aminomethyl group (**3c**), the hydroxyl group at C-2 (**3d**–**3g**), and the double bond at C3–C4 (**3h**,**3i**). Compounds **3j** and **3k** were also obtained under treatment with SOCl_2_.

**Scheme 3:**
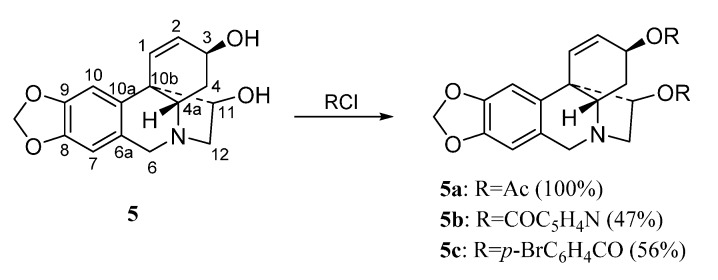
Preparation of derivatives **5a**–**5c** from 11-hydroxyvittatine (**5**).

**Scheme 4:**
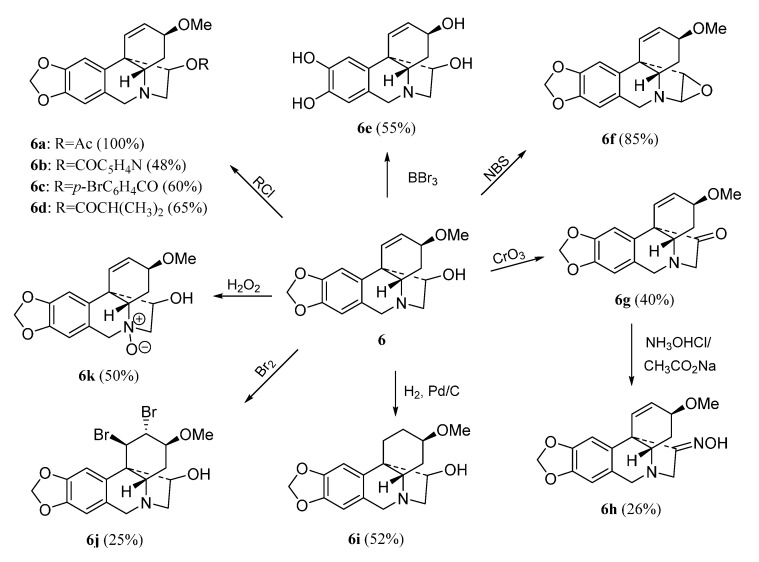
Preparation of derivatives **6a**–**6k** from haemanthamine (**6**).

**Scheme 5:**
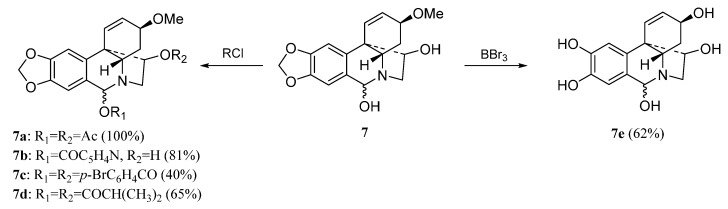
Preparation of derivatives **7a**–**7e** from haemanthidine (**7**).

The haemanthamine-type derivatives are shown in Scheme 3, Scheme 4 and Scheme 5 [6]. These compounds have been prepared from the alkaloids 11-hydroxyvittatine **5** (compounds **5a**–**5c**, Scheme 3), haemanthamine **6** (derivatives **6a**–**6k**, Scheme 4) and haemanthidine **7** (compounds **7a**–**7e**, Scheme 5). Similarly, the transformations carried out with compound **3** were performed with these alkaloids.

All compounds were tested for their antiproliferative activity against the human solid tumor cell lines A2780 (ovary), SW1573 (lung), T-47D (breast) and WiDr (colon) [17]. The data on antiproliferative activity shown in Table 1 allows a classification of the compounds in three groups.

**Table 1:** *In vitro* antiproliferative activity against human solid tumor cells ^a^.

Compound	A2780	SW1573	T47-D	WiDr
**1**	≥100	≥100	≥100	≥100
**2**	8.3 ± 0.5	4.3 ± 0.7	6.5 ± 2.0	9.1 ± 1.0
**2a**	3.4 ± 1.0	3.9 ± 0.7	8.8 ± 1.0	7.5 ± 2.0
**2b**	75.2 ± 25.0	≥100	≥100	≥100
**3**	16.5 ± 10.0	12.5 ± 7.0	52.9 ± 14.0	39.1 ± 20.0
**3a**	41.1 ± 3.0	90.3 ± 11.0	≥ 100	≥100
**3b**	16.8 ± 7.0	17.3 ± 9.0	26.7 ± 10.0	29.1 ± 11.0
**3c**	≥100	≥100	≥100	≥100
**3d**	58.7 ± 7.0	91.5 ± 10.0	≥100	≥100
**3e**	≥100	≥100	≥100	≥100
**3f**	≥100	≥100	≥100	≥100
**3g**	14.6 ± 8.0	25.0 ± 6.0	≥100	≥100
**3h**	≥100	≥100	≥100	≥100
**3i**	67.2 ± 13.0	≥100	≥100	≥100
**3j**	≥100	≥100	≥100	≥100
**3k**	54.9 ± 24.0	≥100	≥100	≥100
**4**	≥100	≥100	≥100	≥100
**5**	21.0 ± 2.0	16.9 ± 4.0	12.5 ± 9.0	21.1 ± 6.0
**5a**	≥100	≥100	≥100	≥100
**5b**	35.9 ± 5.0	34.8 ± 4.0	51.1 ± 3.0	53.8 ± 3.0
**5c**	100	100	100	100
**6**	0.68 ± 0.2	2.1 ± 2.0	0.87 ± 0.4	1.2 ± 0.5
**6a**	≥100	≥100	≥100	≥100
**6b**	27.2 ± 10.0	29.5 ± 12.0	72.3 ± 24.0	63.3 ± 35.0
**6c**	19.1 ± 1.0	21.9 ± 4.0	46.1 ± 30.0	32.8 ± 22.0
**6d**	≥100	≥100	≥100	≥100
**6e**	≥100	≥100	≥100	≥100
**6f**	≥100	≥100	≥100	≥100
**6g**	1.5 ± 0.1	2.7 ± 0.1	4.4 ± 1.5	3.5 ± 2.0
**6h**	33.2 ± 2.0	39.1 ± 20.0	78.6 ± 24.0	67.4 ± 34.0
**6i**	31.4 ± 7.0	29.8 ± 3.0	≥100	58.9 ± 12.0
**6j**	≥100	≥100	≥100	≥100
**6k**	27.2 ± 5.0	22.0 ± 20.0	≥100	≥100
**7**	1.5 ± 0.1	2.0 ± 1.0	1.8 ± 1.0	2.7 ± 2.0
**7a**	≥100	≥100	≥100	≥100
**7b**	6.9 ± 1.0	4.5 ± 0.7	8.2 ± 1.2	10.1 ± 0.5
**7c**	≥100	≥100	≥100	≥100
**7d**	≥100	≥100	≥100	≥100
**7e**	≥100	≥100	≥100	≥100

^a^ Values representing GI_50_ are given in µM and are means of two to three experiments.

A first group is formed with the inactive compounds (GI_50_ values ≥ 100 µM). Tazettine (**1**) and vittatine (**4**) belong to this group. The second group includes compounds with GI_50_ values in the range of 10–100 µM, indicating a moderate activity. Alkaloids hippeastrine (**3**) and 11-hydroxyvittatine (**5**) are found in this group. The last and smallest group is composed of those products with GI_50_ ≤ 10 µM, being the most active alkaloids, haemanthamine (**6**) and haemanthidine (**7**), together with the montanine-type alkaloid pancracine (**2**).

Some physicochemical descriptors (MW, LogP, H-bond donors, H-bond acceptors, Rotable bonds and TPSA) of all tested compounds were calculated using Molinspiration Cheminformatics software (2014) and the corresponding values are included in Table 2.

**Table 2:** Physicochemical descriptors ^a,b^.

Compound	MW	LogP	H-Bond Donors	H-Bond Acceptors	Rotable Bonds	TPSA
**1**	331	1.53	1	6	1	60.40
**2**	287	0.54	2	5	1	62.16
**2a**	319	2.39	0	4	1	30.94
**2b**	349	2.55	0	5	2	40.17
**3**	315	1.23	1	6	0	68.24
**3a**	303	0.37	3	6	0	90.23
**3b**	314	0.59	2	6	0	71.03
**3c**	314	−2.91	1	6	0	68.01
**3d**	357	1.93	0	7	2	74.32
**3e**	420	2.37	0	8	3	87.21
**3f**	498	4.47	0	7	3	74.32
**3g**	313	1.04	0	6	0	65.08
**3h**	317	1.41	1	6	0	68.24
**3i**	475	2.29	1	6	0	68.24
**3j**	297	2.12	0	5	0	48.01
**3k**	333	2.60	0	5	0	48.01
**4**	271	1.59	1	4	0	41.93
**5**	287	0.67	2	5	0	62.16
**5a**	371	2.08	0	7	4	74.32
**5b**	497	2.38	0	9	6	100.10
**5c**	653	7.15	0	7	6	74.32
**6**	301	1.29	1	5	1	51.17
**6a**	343	1.99	0	6	3	57.25
**6b**	406	2.14	0	7	4	70.14
**6c**	484	4.52	0	6	4	57.25
**6d**	383	3.46	0	6	4	57.25
**6e**	275	−0.19	4	5	0	84.15
**6f**	299	1.74	0	5	1	43.47
**6g**	299	1.10	0	5	1	48.01
**6h**	314	1.55	1	6	1	63.53
**6i**	301	1.29	1	5	1	51.17
**6j**	461	2.22	1	5	1	51.17
**6k**	317	1.25	1	6	1	65.00
**7**	317	0.83	2	6	1	71.40
**7a**	401	2.23	0	8	5	83.55
**7b**	422	1.68	1	8	4	90.37
**7c**	683	7.30	0	8	7	83.55
**7d**	481	5.17	0	8	7	83.55
**7e**	291	−0.65	5	6	0	104.38

^a^ Values were calculated using Molinspiration Cheminformatics software (Molinspiration, Slovensky Grob, Slovak Republic, 2015, http://www.molinspiration.com); ^b^ Optimal range MW < 500, LogP < 5, H-bond donors < 5, H-bond acceptors < 10, Rotable bonds < 5, TPSA < 140.

With respect to the antiproliferative activity of tazzetine (**1**), haemanthamine (**6**), and haemanthidine (**7**), our results are consistent with those obtained by Evidente *et al.* [16] for their antiproliferative activity against six different cancer cell lines (A549, OE21, Hs683, U373, SKMEL and B16F10). Thus tazzetine also turned out to be inactive, and haemanthamine (**6**) and haemanthidine (**7**) had IC_50_ values ranging from 3.1 to 8.5 µM.

From the results of antiproliferative activity some structure-activity relationships can be outlined. The replacement of the hydroxyl groups in the E ring of pancracine (**2**) by a chlorine and a methoxy group (**2a**), respectively, produced a similar result but the introduction of a methoxy group at C-6 (**2b**) reduced the antiproliferative activity. Since **2a** and **2b** have similar Log P values, the steric hindrance at C-6 seems an important factor for the activity of this series of compounds.

Regarding to the alkaloids of the hippeastrine series, all modifications made at the hydroxyl group of hippeastrine (**3**) (derivatives **3d**–**3g**) produced a significant loss of the activity, indicating the importance of a hydrogen-bond-donor (HBD) at C-2. The role of the C3-C4 double bond was evident since inactive derivatives **3h** and **3i** were obtained under hydrogenation or bromination. Another important group is the methylendioxy because when this group was converted into the corresponding aromatic diol **3a**, the activity decreased drastically. On the other hand, the presence of the lactone ring is not essential for the activity, thus when the lactone moiety was transformed into the lactam (derivative **3b**), similar activities were obtained.

Comparison of the antiproliferative activities of the natural alkaloids vittatine (**4**), 11-hydroxyvittatine (**5**), haemanthamine (**6**) and haemanthidine (**7**) indicate how important are for the activity the presence of a methoxy group at C-3 together an hydroxyl group at C-11.

These facts were confirmed with the preparation of derivatives **6a**–**f**. Furthermore compounds **5**, **6** and **7** have lower LogP than the inactive compound **4**. The obtention of the inactive derivatives **6i** and **6j** shows the importance of the double bond at C1-C2 for the activity. Removal of the methylendioxy group also led to a less active compound (**6k**), indicating that this group is also important for the haemanthamine series. Haemanthidine **7**, which possesses a hydroxyl group at C-6, can be considered as active as haemanthamine **6**. The acylation of the hydroxyl groups at C-6 and C-11 produces a loss of antiproliferative activity (**7a**, **7c**, **7d**) but compound **7b** having a free hydroxyl at C-11 and a nicotinoyl group at C-6.

Since most of the alkaloids evaluated for antiproliferative activity were also previously evaluated for antimalarial activity [6,7], a comparative antiproliferative *vs.* antimalarial SAR study is included. For antimalarial activity the natural alkaloids tazzettine (**1**) and vittatine (**4**) were active against *Plasmodium falciparum*, while they were inactive for antiproliferative activity. With respect to the compounds related to pancracine (**2**), we obtained identical SAR for both activities. Regarding the derivatives **3a**–**3k**, similar SAR were determined for the modifications on the hydroxyl group at C-2, and on the double bond C-3–C-4. The aromatic diol **3a** obtained from the transformation of the methylendioxy group resulted inactive for antiproliferative activity, but it showed good antimalarial activity. The same behavior was detected for compound **6e**. In the derivatives obtained from the diol **5** all diesterified compounds resulted inactive for antiproliferative activity but compound **5b**, with two nicotinoyl groups, which showed high antimalarial activity. For the derivatives obtained from **6**, all modifications carried out on the hydroxyl group at C-11 led to a loss of antimalarial and antiproliferative activity, while the hydrogenation of the double bond produced opposite results; compound **6i** resulted inactive for antiproliferative activity and had antiplasmodial activity. Finally, similar SAR for antiproliferative and antimalarial activities were obtained for the derivatives **7a**–**7d**.

## 3. Experimental Section

### 3.1. Natural and Semisynthetic Amaryllidaceae Alkaloids

The natural alkaloids Tazettine (**1**) (3 mg), pancracine (**2**) (17 mg), hippeastrine **3** (1.35 g), vittatine **4** (8 mg), 11-hydroxyvittatine **5** (123 mg), haemanthamine **6** (2.01 g) and haemanthidine **7** (360 mg) were isolated from *Pancratium canariense* as describe in reference [15]. Derivatives **2a** and **2b** were prepared according to the procedure described in reference [17]. Homolycorine-type derivatives **3a**–**3k** were synthesized following the reference [7] while the haemathamine-type derivatives were obtained as describe in reference [6].

### 3.2. Antiproliferative Assay

Growth inhibition and cytotoxicity against the human solid tumor lines A2780 (ovary), SW1573 (lung), T-47D (breast) and WiDr (colon) was screened using the sulforhodamine B (SRB) assay described in reference [18]. Cells were inoculated at densities of 7000 (A2780), 6000 (SW1573), 15,000 (T-47D) and 10,000 (WiDr) cells per well, based on their doubling times. Pure compounds were initially dissolved in DMSO at 400 times the desired final maximum test concentration (100 µM). Control cells were exposed to an equivalent concentration of DMSO. Each agent was tested in duplicate at five different tenfold dilutions. Drug incubation times were 48 h, after which cells were precipitated with 25 µL ice-cold 50% (*w*/*v*) trichloroacetic acid and fixed for 60 min at 4 °C. Then the SRB assay was performed. The optical density (OD) of each cell was measured at 490 nm using a Bio-Tek’s Elx800 NB 96-well plate reader. The percentage growth was calculated at each of the drug concentration levels based on the difference in OD at the start and end of drug exposure. Values were corrected for background OD from wells only containing medium. The resulting biological activities are expressed as GI_50_, the concentration of compound responsible of a 50% growth inhibition, and are shown in Table 1.

## 4. Conclusions

In conclusion, a set of diverse Amaryllidaceae alkaloids with different skeletons has been tested for antiproliferative activity. The compounds belonging to the pancracine and haemantine series were the most active. From the obtained result the key structural requirements for each series were outlined. The best antiproliferative activities were achieved with the natural alkaloids **6** and **7** and also with the derivatives **6g** and **7b**. The physicochemical descriptors (Table 2) of these compounds do not violate the optimal requirements for druggability, which suggests that these alkaloids are promising lead compounds for further research.
